# Address

**Published:** 1863-03

**Authors:** J. Mansfield


### ADDRESS,

(Read before the Michigan State Dental Association.)

BY J. MANSFIELD.

Mr. President and Gentlemen:—I am no essayist and regret that you did not choose a more able and proper person to write an essay on the treatment of exposed nerve. I will give you my experience for the last twenty years. I expect to differ from most of you on the subject, as I do from most of the writers that have written on it. But I think we should be honest and frank with ourselves and profession, not say or believe a thing because some one else thinks or says so; but take our own experience and use a little thought and judgment of our own and weigh the matter in our minds and see if the writer has not a theory without the practical part.

I have often tried to save an exposed nerve by various treatments and have almost invariably found that my labor was in vain, and in young persons I think I might say that I have failed in almost every case, and my practice is now to destroy the nerve before filling. If I attempt to fill over the nerve the tooth sooner or later becomes sore, the alveolus inflamed, the nerve dies and you are obliged to remove the filling and extripate the dead nerve and treat the tooth and gum for alveolar abscess, thus losing much labor or charging your patient two prices for one operation and leaving the tooth in a worse condition than you would if you had destroyed the nerve in the first place. You will fail in nine cases out of ten if you attempt to save an exposed nerve. I have tried various applications for destroying the nerve and now use equal parts of arsenic and morphine made into a thin paste with creosote, filling it with small bits of cotton twine, cut as fine as you can cut it, corking the phial tight for one or two weeks, then leave the cork out and let the creosote evaporate. My object in preparing it in this way is to prevent it from escaping and getting on to the gum, and to use a lesser quantity of the arsenic. The least you can use the better. It is an error to apply creosote and arsenic in a moist or thin state, because the creosote is absorbed readily by the dentine, taking a portion of the arsenic with it, producing inflammation of the dentine and often of the surrounding parts. Clean the cavity well before putting in the arsenic, and I prefer to uncap the nerve and make it bleed before making the application. If you uncap the nerve, and cause it to bleed the patient will not experience much pain, and the less pain you give the less inflammation and soreness you may expect to find in the tooth and adjacent parts when you prepare the tooth for filling. I let the arsenic remain from eight to twenty-four hours. In young persons you will find the dentine more porous and readily absorbs the arsenic, thus requiring more caution on the part of the operator. Then remove the arsenic, cleansing the cavity, being careful to remove every particle of the arsenic. If the tooth is not too sore extract the nerve, if you are not able to do that cut it to pieces as much as you can. If the tooth is sore and the patient nervous omit it for a few days and apply creosote, stopping the cavity with wax or cotton, changing it every day or two as the case may require. I seldom fill in less than two or three weeks after destroying the nerve. If you fill in less time you may have a discolored and sensitive tooth. The nerve at the apex of the tooth, if it is not entirely cut off and healed before filling, the blood will discharge into the nerve cavity and diffuse itself into every pore of the tooth, discoloring it and producing inflammation of the tooth and the alveolus; thus displeasing your patient and being an eye-sore to yourself.

NILES, January, 1863.

